# Lessons from 5 Years of Routine Whole-Genome Sequencing for Epidemiologic Surveillance of Shiga Toxin–Producing *Escherichia coli*, France, 2018–2022

**DOI:** 10.3201/eid3113.241950

**Published:** 2025-05

**Authors:** Gabrielle Jones, Carolina Silva Nodari, Laëtitia Fabre, Henriette de Valk, Harold Noel, Aurélie Cointe, Stéphane Bonacorsi, François-Xavier Weill, Yann Le Strat

**Affiliations:** Santé publique France, Saint-Maurice, France (G. Jones, H. de Valk, H. Noel, Y. Le Strat); Institut Pasteur, Université Paris Cité, Paris, France (C. Silva Nodari, L. Fabre, F.-X. Weill); Centre hospitalier universitaire Robert Debré, Assistance Publique–Hôpitaux de Paris, Paris (A. Cointe, S. Bonacorsi)

**Keywords:** Shiga toxin–producing Escherichia coli, bacteria, STEC, whole-genome sequencing, epidemiologic surveillance, hierarchical clustering, cluster detection, France

## Abstract

Whole-genome sequencing (WGS) is routine for surveillance of Shiga toxin–producing *Escherichia coli* human isolates in France. Protocols use EnteroBase hierarchical clustering at <5 allelic differences (HC5) as screening for cluster detection. We assessed current implementation after 5 years for 1,002 sequenced isolates. From genomic distances of serotypes O26:H11, O157:H7, O80:H2, and O103:H2, we determined statistical thresholds for cluster determination and compared those with HC5 clusters. Thresholds varied by serotype, 5–16 allelic distances and 15–20 single-nucleotide polymorphisms, showing limits of a single-threshold approach. We confirmed validity of HC5 screening for 3 serotypes because statistical thresholds had limited effect on isolate clustering (high sensitivity and specificity). For O80:H2, results suggest that HC5 is less reliable, and other approaches should be explored. Public health officials should regularly assess WGS used for Shiga toxin–producing *E. coli* surveillance to account for serotype and genomic evolution and to interpret WGS-linked isolates in light of epidemiologic data.

Shiga toxin–producing *Escherichia coli* (STEC) are responsible for a spectrum of disease that ranges from self-resolving diarrhea to bloody diarrhea and severe complications, including hemolytic uremic syndrome (HUS). STEC continues to be a public health risk, and although infections are largely sporadic, STEC has substantial outbreak potential (*1*–*3*). Therefore, surveillance and outbreak detection remain public health priorities (*4*). Advances in STEC detection and typing methods over the past decade, including the expansion of culture-independent diagnostic tests and whole-genome sequencing (WGS), have affected diagnostic approaches, expanded knowledge of pathogenicity, informed source attribution, improved outbreak detection capacities, and guided surveillance protocols (*5*–*9*).

Advantages of implementing WGS for epidemiologic surveillance are widely documented. WGS is the primary method of foodborne pathogen surveillance and outbreak detection in numerous countries in Europe and North America (*5*,*10*–*12*). Diverse studies have confirmed superiority of WGS for cluster determination, shown validation of thresholds used for cluster detection in surveillance protocols, and described WGS-linked isolates in light of epidemiologic data (*6*,*11*,*13*–*19*). WGS improves outbreak detection and investigation capacity by providing more timely cluster detection and discriminatory case definitions and detecting geographically and temporally diffuse clusters. Such studies are essential for guiding the international adoption of widespread use of WGS for disease surveillance and outbreak detection. However, surveillance systems and epidemiologic context differ between countries, and multiple WGS approaches are possible for isolate comparison (*6*,*9*,*15*,*20*). Therefore, assessing the implementation of WGS for epidemiologic surveillance specific to a given pathogen and country is vital.

WGS was implemented in France for STEC surveillance in early 2017 (*3*). Surveillance uses the EnteroBase (https://enterobase.warwick.ac.uk) core-genome multilocus sequence typing (cgMLST) hierarchical clustering method (HierCC) for *E. coli* as an initial screening step for cluster detection at <5 allelic differences (HC5) (*21*–*23*). HC5 clusters are confirmed by core-genome single-nucleotide polymorphism (SNP) tree analysis.

On the basis of 5 years (2018–2022) of retrospective data available from STEC surveillance, this study aimed to assess implementation of WGS for cluster detection protocols in France. The first objective was to apply statistical approaches to pairwise allelic distance (AD) and SNP distance data to evaluate whether thresholds could be determined to define genomic proximity. The second objective was to assess the performance of those statistical thresholds compared with HC5. Finally, we described genomic distance data by considering HC5 and associated epidemiologic data.

## Methods

### STEC Surveillance and Cluster Detection in France

STEC surveillance and outbreak detection in France rely on 2 previously described parallel voluntary systems: epidemiologic surveillance of HUS in children <15 years of age, coordinated at the national level by the food and waterborne disease surveillance and outbreak investigation unit at Santé publique France (French public health agency, https://www.santepubliquefrance.fr); and microbiological surveillance coordinated by the National Reference Center for *E. coli*, *Shigella*, *Salmonella* (NRC-ESS) and its associated NRC at Robert Debré hospital, Paris (NRC-RD) (*1*,*3*). Epidemiologists at regional offices of Santé Publique France can also contribute to investigations but are not dedicated to foodborne disease surveillance. Santé publique France links epidemiologic data from pediatric STEC-HUS surveillance and epidemiologic investigations to WGS data, generating a consolidated anonymous dataset for annual surveillance reports (*3*).

A cluster is typically defined as cases grouped in space, time, or both. An outbreak defines cases for which an epidemiologic link is identified. A microbiological cluster defines isolates grouped on the basis of an established typing method: phenotypic serogroup and serotype or genomic typing using cgMLST or SNP analysis. Cluster detection in France relies on pediatric HUS notifications and microbiological data (Appendix 1).

In current WGS protocols, STEC genomic data are submitted to EnteroBase with limited metadata (isolate source, e.g., human, food; sampling year; and country). The cgMLST and HierCC schemes implemented in that platform assist in identification of genomic clusters (Appendix 1) (*21*). The platform uses multilevel, static, cluster assignments of bacterial genomes to describe genetic diversity (*23*). At the French NRC, the HC5 level of the HierCC scheme is used for screening of genomic relatedness for epidemiologic purposes. If necessary, particularly for HC5s that persist over time, an additional SNP analysis using the EnteroBase pipeline serves as a confirmatory step. Epidemiologists assess cluster characteristics (size, space-time distribution, clinical severity, case-patient characteristics) to decide whether investigations should be initiated. Decisions to investigate small (<5 isolates) or temporally dispersed WGS clusters also depend on availability of human resources.

### Study Data

We included STEC isolates sequenced at the NRC-ESS and uploaded to EnteroBase as part of routine WGS data analysis from January 1, 2018–December 31, 2022. We considered isolates from the same patient as duplicates and excluded those if sampling dates were <2 weeks apart and WGS identified the same strain. We restricted analyses to 4 serotypes with sufficient historical data: O26:H11 (n = 478), O80:H2 (n = 226), O157:H7 (n = 223), and O103:H2 (n = 75). We conducted all data management and statistical analyses in R version 4.2 (The R Project for Statistical Computing, https://www.r-project.org).

The assembled short-read data for the list of genomes are available from EnteroBase (https://enterobase.warwick.ac.uk/species/ecoli/search_strains?query=workspace:127168) (Appendix 2 Table 1). Short-read sequences are available at the European Nucleotide Archive (https://www.ebi.ac.uk/ena/browser/home; project no. PRJEB50273.

### Allelic and SNP Distance Distributions

We generated pairwise allele and SNP distance matrices for each serotype. We extracted the cgMLST allelic profiles from EnteroBase and excluded alleles if they were missing from >5% of isolates within a given serotype (2 excluded from O157:H7 AD matrices). We calculated AD from allelic profiles on the basis of the number of mismatched loci and determined SNP distances on a recombination-free multisequence alignment of the core genome of each studied serotype (Appendix 1).

We merged isolate characteristics (sampling date, HC2–HC50, epidemiologic data) from consolidated surveillance datasets with the AD and SNP matrices by using a unique anonymous identifier from the NRC-ESS. For each serotype, we plotted overall distribution of pairwise AD and SNP. We censured data at 50 AD and SNP distance for statistical analysis and primary graphical representations.

### Determination of Statistical Genomic Distance Thresholds

We applied a mixture of distributions approach to test whether statistical thresholds to describe genomic proximity of isolates could be determined. Mixture of distributions is a classic statistical approach for determining thresholds from continuous data distributions, such as seroprevalence data (*24*). We used the mixR package in R, which determines the best fit to continuous data from several distribution families and selects the optimal number of components for the mixture model on the basis of the lowest value of the Bayesian information criterion (*25*). The underlying hypothesis was that outbreak-related isolates have smaller pairwise AD and SNP distance. For each pair of isolates, the probability of belonging to the first distribution (comprising the smallest genomic distances) is calculated and plotted according to AD and SNP distance. We set a threshold as the AD or SNP distance at which the probability of belonging to the first distribution was >50%.

### Comparison of Genomic Distributions and Statistical Thresholds to HC5 Clusters

Although isolates are assigned to HC on the basis of AD, HC does not strictly translate to AD because of the multilevel clustering approach, which defines that when AD at a given level is equal, the genome is assigned to the oldest HC value (*23*). For example, isolates assigned to a given HC5 or HC10 cluster are not all within 5 or 10 AD of each other. Therefore, assessing the observed genomic distance distributions and performance of statistically defined thresholds in relation to HC5 clusters is necessary. We calculated sensitivity and specificity of statistically determined thresholds compared with HC5.

We also assessed the relationship between time and genomic distances within HC5 clusters. We studied time in categories constituted on equal distribution of isolates and as a continuous variable (days) by using a multivariable fractional polynomial (MFP) linear regression. To assess concordance between HC5 and SNP analysis as a confirmatory step for cluster determination, we visualized HC5 clusters (>4 isolates) and year (all isolates) into generated SNP-based maximum-likelihood trees by using iTOL (https://itol.embl.de) (*26*) (Appendix 1).

### Genomic and Epidemiologic Characteristics of HC5 Clusters

We assessed characteristics for each HC5 cluster, including genomic distance range, number of isolates, temporal distribution, geographic distribution (same administrative department or region, multiple regions), and epidemiologic link. Epidemiologic links included clusters of household transmission and single patients (isolates sampled >15 days apart), isolates with a confirmed or suspected outbreak link, and isolates for which the link was unable to be determined from investigations.

## Results

### Pairwise Distance Distributions

**Figure 1:**
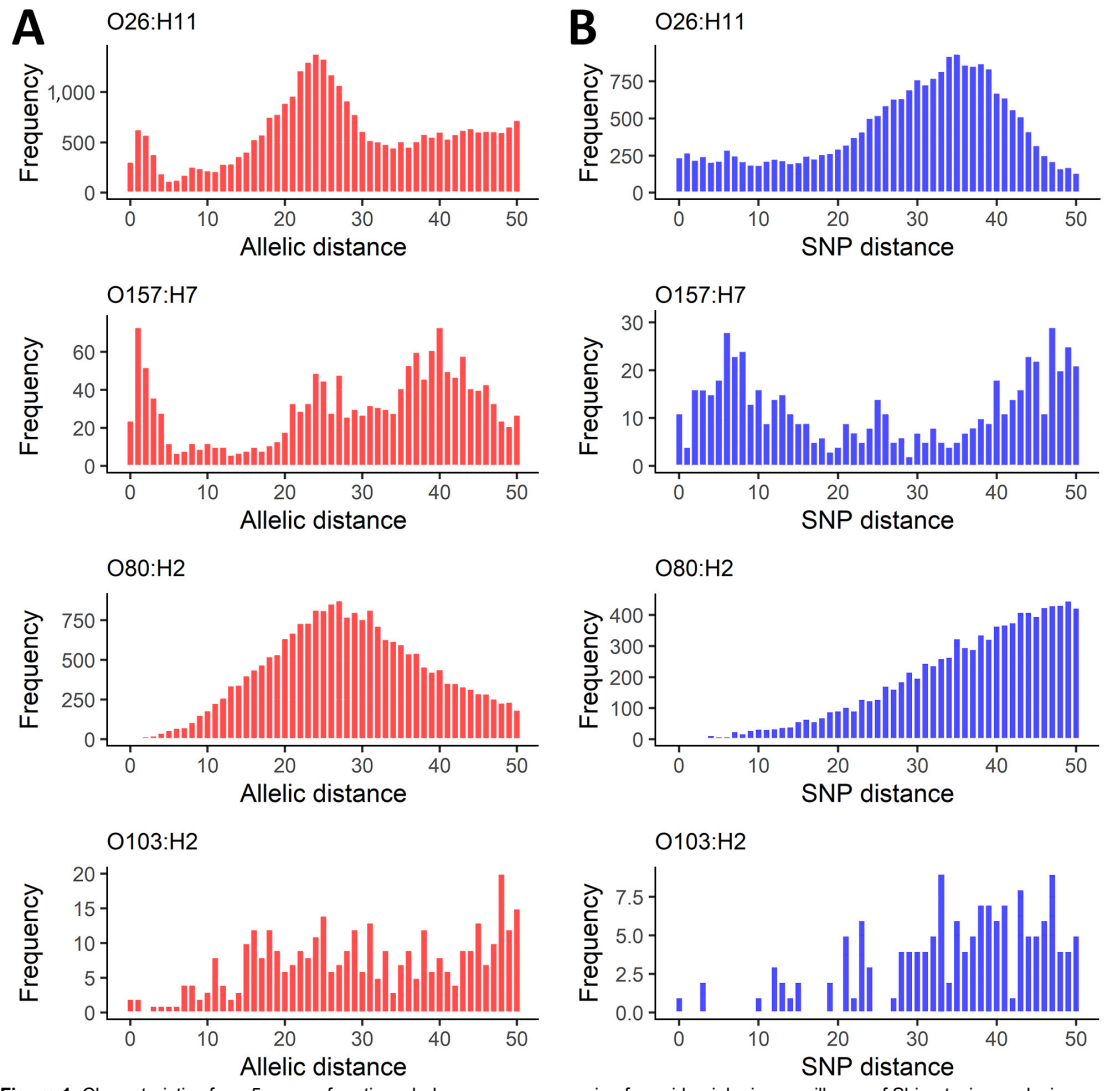
Characteristics from 5 years of routine whole-genome sequencing for epidemiologic surveillance of Shiga toxin–producing *Escherichia coli*, France, 2018–2022. A) Distribution of pairwise allelic distances; B) SNP distances, censured at 50. Shiga toxin–producing *Escherichia coli* serotypes are shown for each panel. SNP, single-nucleotide polymorphism.

Genomic distance distributions varied by serotype (Appendix 1 Figure 1, panels A, B). For O26:H11 and O157:H7, we observed a peak at 0–5 AD (Figure 1, panel A). Conversely, fewer O80:H2 isolate pairs had shorter AD, and we observed no similar peak but noted a normal distribution. Few O103:H2 isolate pairs had AD <10. The O26:H11 SNP distance distribution showed a plateau from 1 to 20 SNPs (Figure 1, panel B). For O157:H7, we observed a peak of 0–20 for pairwise SNP distances. The SNP distance distribution for O80:H2 showed a sloping increase, and few isolate pairs had <10 SNP distance. The O103:H2 SNP difference distribution was sparse, limiting description of specific characteristics.

### Determination of Statistical Thresholds

**Figure 2:**
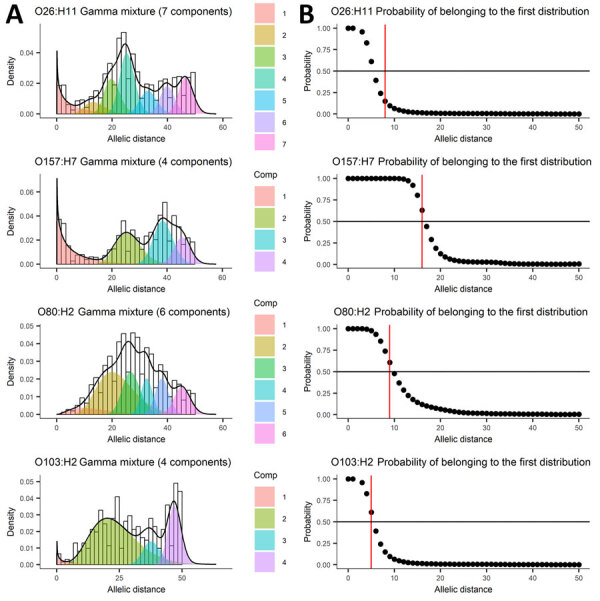
Mixture of distributions model applied to allelic distance data from 5 years of routine whole-genome sequencing for epidemiologic surveillance of Shiga toxin–producing *Escherichia coli*, France, 2018–2022. A) Number of components fit to the data distribution; B) threshold represented as the probability of belonging to the first distribution. Shiga toxin–producing *Escherichia coli* serotypes are shown for each panel. Black line indicates global estimated density; black circles, probability of belonging to first distribution for each observed allelic or single-nucleotide polymorphism distance; red line, largest allelic or single-nucleotide polymorphism distance that has a 50% probability of belonging to the first distribution. Comp, component.

**Figure 3:**
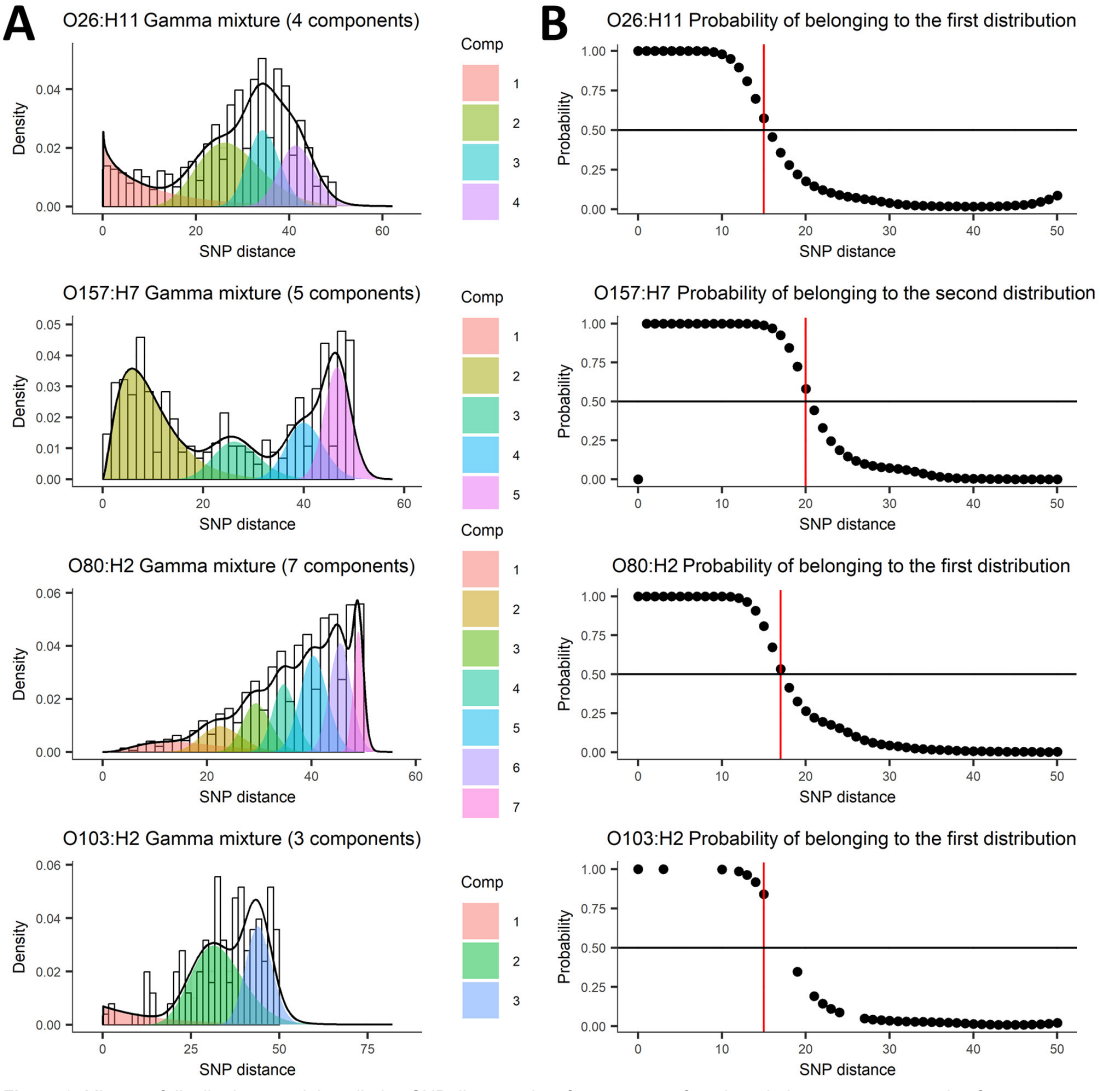
Mixture of distributions model applied to SNP distance data from 5 years of routine whole-genome sequencing for epidemiologic surveillance of Shiga toxin–producing *Escherichia coli*, France, 2018–2022. A) Number of components fit to the data distribution; B) threshold represented as a probability of belonging to the first or second distribution. Shiga toxin–producing *Escherichia coli* serotypes are shown for each panel. Comp, component; SNP, single-nucleotide polymorphism.

The mixture of distributions model retained the gamma distribution for determination of both AD and SNP distance thresholds. The number of components fitting the genomic distance distributions in the final model varied by serotype (Figure 2, panel A; Figure 3, panel A). The AD statistical thresholds were <8 AD for O26:H11, <16 AD for O157:H7, <9 AD for O80:H2, and <5 AD for O103:H2 (Figure 2, panel B). The SNP distance statistical thresholds were <15 SNP for O26:H11, <20 SNP for O157:H7, <17 SNP for O80:H2, and <15 SNP for O103:H2 (Figure 3, panel B). For O157:H7 SNP distances, we determined the threshold from the probability of belonging to the second distribution, because the first distribution was at 0, with mean and SD close to 0. Although we determined a threshold for O103:H2, the result was less robust because of the small number of pairwise isolates, particularly at shorter genomic distances.

### Genomic Distance Distributions within HC5

The number of HC5 clusters increased with serotype frequency: 6 for O103:H2, 19 for O157:H7, 23 for O80:H2, and 39 for O26:H11. The AD and SNP distance distributions observed in HC5 clusters varied within and between serotypes (Appendix 1 Figure 2). Applying statistically determined thresholds, all HC5 cluster isolates were under the AD threshold for serotypes O103:H2 and O157:H7. Only O103:H2 HC5 cluster isolates were under the SNP distance threshold (Appendix 1 Figure 2). A greater number of O26:H11 and O80:H2 HC5 clusters contained isolate pairs surpassing statistical thresholds.

Sensitivity and specificity of the statistical thresholds compared with HC5 clusters varied between serotypes (Table). For O157:H7 and O103:H2, the statistical thresholds had high sensitivity (>99%) and specificity (83%–100%). For O26:H11, sensitivity was close to 100%, and specificity was 73% for AD threshold and 88% for SNP threshold. Finally, for O80:H2, although the mixture of distributions determined a statistical threshold, specificity was poor for both AD (34%) and SNP (35%) thresholds.

**Table:** Sensitivity and specificity of statistically determined allelic and SNP distance thresholds from 5 years of routine whole-genome sequencing for epidemiologic surveillance of Shiga toxin–producing *Escherichia coli*, France, 2018–2022*

Serotype	Allelic distance				SNP distance		
	Threshold, no. alleles	Sensitivity, %	Specificity, %		Threshold, no. SNPs	Sensitivity, %	Specificity, %
O26:H11	<8	99.9	73.1		<15	99.6	87.8
O157:H7	<16	99.7	99.6		<20	99.9	96.7
O80:H2	<9	99.8	33.6		<17	99.6	35.1
O103:H2	<5	100	83.3		<15	99.0	100

*SNP, single-nucleotide polymorphism.

### Genomic Distance Distributions within HC5 Clusters as a Function of Time

**Figure 4:**
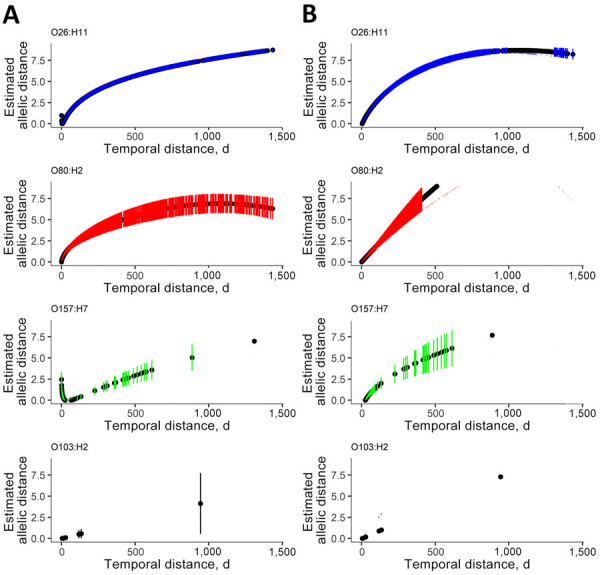
Regression from hierarchical clustering at a threshold of 5 allelic differences from 5 years of routine whole-genome sequencing for epidemiologic surveillance of Shiga toxin–producing *Escherichia coli*, France, 2018–2022. A) Allelic distance; B) SNP distance. Distances calculated as a function of time in days by multivariable fractional polynomial linear regression. Black circles indicate estimated allelic or SNP distance for each observed temporal distance in days; blue, red, green, and black vertical lines, 95% CIs of the estimated genomic distances for each observed temporal distance in days. SNP, single-nucleotide polymorphism.

With time represented in classes, we observed a slight positive association between AD and HC5 (Appendix 1 Figure 3). MFP regression integrated time as a continuous variable and confirmed a linear relationship with AD for all serotypes, but with varied strength of association (Figure 4, panel A). Of note, we found a negative association between AD and time observed for O26:H11 and O157:H7 at the smallest temporal distances (<5 days) and then a positive linear relationship as temporal distance increased. For O103:H2, the relationship was linear, but the number of HC5 clusters was small, and the maximum temporal distance was comparatively short (≈100 days). Analysis with SNP distance yielded similar results as AD, with 1 distinct difference: MFP regression did not identify the same negative association at small temporal distances for O26:H11 and O157:H7 (Figure 4, panel B; Appendix 1 Figure 4).

### Concordance between HC5 and SNP

**Figure 5:**
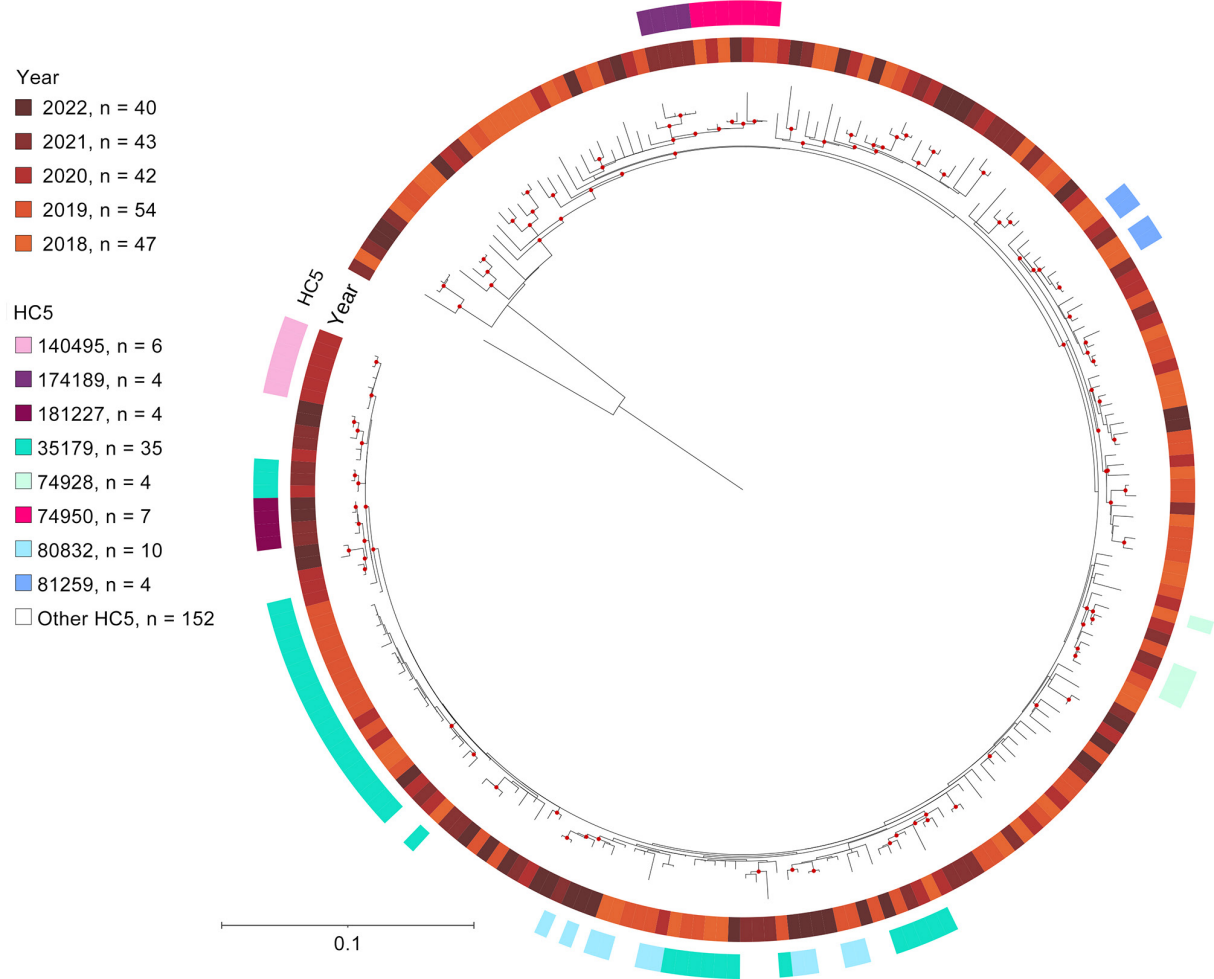
Single-nucleotide polymorphism–based maximum likelihood phylogenetic tree of 226 080:H2 isolates from 5 years of routine whole-genome sequencing for epidemiologic surveillance of Shiga toxin–producing *Escherichia coli*, France, 2018–2022. Tree was built based on the sequence alignment of 3,949 single-nucleotide variant sites of the recombination-free core genome of *E. coli* strain MOD1-EC6881 (GenBank accession no. GCF_002520045.1). Tree was midpoint-rooted and visualized with iTOL (https://itol.embl.de). Bootstrap support values >90% are indicated with red dots on the branches. Branch lengths and corresponding scale bar indicate numbers of single-nucleotide polymorphisms per base of the final alignment. HC5, hierarchical clustering at a threshold of 5 allelic differences.

SNP analysis generally confirmed HC5 clusters for all serotypes except O80:H2 (Appendix 1 Figures 5–7). For O80:H2, although SNP distance confirmed clustering for some HC5s, for others, such as HC5_35789 and HC5_80832, HC5 was not predictive of SNP clustering because genomes belonging to the same HC5 were dispersed in the phylogenetic tree (Figure 5).

### Genomic Distance and Epidemiologic Characteristics of HC5 Clusters

Because HC5 informed cluster detection and guided epidemiologic investigations during the study period, data are not independent. However, examining differences in genomic distance in light of epidemiologic characteristics of HC5 clusters is of interest.

We identified 87 HC5 clusters (>2 isolates) comprising 449 isolates over the study period. Most (81/87; 93%) clusters comprised 2–10 isolates; 80% (70/87) of the HC5 clusters comprised 2–4 isolates, and 13% (11/87) comprised 5–10 isolates (Appendix 2 Table 2).

For the 81 clusters with 2–10 isolates, 58 (72%) comprised isolates with sampling dates within 1 year of each other. Twenty (25%) clusters had a duration of 1–2 years, and 4 (5%) clusters had a duration >3 years. Of the 6 HC5 clusters with >10 isolates, 4 lasted >3 years and 2 had isolates sampled within 3-month periods. Geographic distribution expanded with cluster size. All HC5 clusters within the same administrative department had <5 isolates, and all clusters within the same region had <10 isolates.

For clusters of 2–10 isolates, median AD ranged from 0–5 (O103:H2) to 0–15 (O26:H11), and median SNP distance ranged from 0–14 (O103:H2) to 5–28 (O157:H7). For the 6 larger clusters (>10 isolates), 2 were point-source outbreaks (O157:H7 HC5_116498 [suspected] and O26:H11 HC5_190514 [confirmed]), with reasonably small median genomic distances: median AD = 1 for both and median SNP distance = 6 for O157:H7 HC5_116498 and 4 for O26:H11 HC5_190514. The 4 other large clusters with isolates sampled over 3–5 years had median AD of 2–10 and median SNP distance of 8–21. We observed the highest median and maximum genomic distances for O80:H2.

We linked 6 HC5 clusters (all <5 isolates) exclusively to household transmission, and we linked 1 cluster to 1 patient. Those median genomic distances were small. Of the additional 27 HC5 clusters that led to epidemiologic investigations of all or some cases (depending on space-time distribution), we identified a confirmed or suspected epidemiologic link for 20 (74%) clusters, corresponding to 146 isolates (15% of the study population) (Appendix 2 Table 2) (*27*–*29*). Those links included 2 persistent O26:H11 clusters (HC5_65006 and HC5_75047) comprising isolates associated with multiple point-source outbreaks and sporadic isolates with no identified epidemiologic link to each other or with previous outbreak sources (*28*,*29*). Within O26:H11 clusters that comprised isolates with documented epidemiologic links to several different point-source outbreaks, the median genomic distances of epidemiologically linked isolates were smaller than those of the overall cluster (Appendix 2 Table 2).

## Discussion

The results of this study describe advantages and challenges of WGS for epidemiologic surveillance of STEC and inform potential adaptations in surveillance protocols in France. In this study, we used pairwise genomic distances to explore the robustness of using WGS-based clustering, particularly the HC5 level of EnteroBase’s HierCC scheme, as a screening threshold for outbreak detection in STEC surveillance in France after 5 years of routine use. We first determined statistical thresholds to define genomic proximity. The heterogeneity of the thresholds across serotypes showed the necessity of verifying the suitability of a given approach strictly on the basis of genomic distance thresholds to all serotypes. Except for O80:H2, we confirmed the validity of using HC5 for a screening step for microbiological cluster determination; applying the statistical thresholds had a limited effect on how isolates grouped compared with HC5.

The O80:H2 genomic distance distributions were visually distinct, with near normal distributions versus multimodal distributions. SNP analysis for O80:H2 showed limited concordance with specific HC5 clusters compared with the other serotypes. Factors influencing genomic diversity, including mutation rate, reservoir, and transmission pathways, may differ for O80:H2 and explain its limited genomic diversity (*30*). The lack of concordance between cgMLST, including HC5, and epidemiologically relevant clusters has also been observed for another pathogenic clone of *E. coli* that exhibits limited genomic diversity, the human-restricted enteric pathogen *Shigella sonnei*, leading to a reliance on high-resolution techniques for surveillance (*31*). That observation suggests O80:H2 cluster determination should rely on SNP-based phylogenies. Such approaches require selection of an appropriate reference isolate and continuous integration of emerging strains into the analysis. Those approaches do not confer the same advantages of cgMLST and the EnteroBase’s HierCC scheme, such as ease of comparing isolates with standardized methodology and nomenclature. Although O80:H2 is in the top 3 serotypes isolated in France since 2015, it is an uncommon hybrid pathotype (STEC/ExPEC [extraintestinal pathogenic *E. coli*]) that emerged in the early 2010s, and its reservoirs remain unclear (*1*,*30*). Indeed, a case–case study comparing characteristics and reported risk factors of *E. coli* O80–infected children with HUS with those infected by other STEC serogroups in France concluded that epidemiologic characteristics of O80:H2-infected pediatric HUS cases differed from O157:H7 and other serotypes (*32*). Also, although O80:H2 was isolated in healthy cattle in France in 2023 and diarrheic calves in Belgium, no outbreaks have been documented in France after epidemiologic investigations (*33*,*34*). Improving cluster discrimination could increase the likelihood of resolving epidemiologic investigations and advancing knowledge on potential sources of contamination and reservoirs.

This study had several limitations related to data availability. Of note, analyses depended on the number of isolates available in surveillance data for France and pertained to 4 primary serotypes. The results suggest that conclusions may differ for other serotypes, and when sufficient isolates are available, expanding the study will be pertinent. Because STEC surveillance in France is voluntary, isolate data are not representative of all STEC in France. Pediatric HUS surveillance data are considered representative (*3*). However, that is not the case for other clinical isolates because patients with more severe illness are more likely to have consultations or be hospitalized and have biological sampling (*35*). Few environmental, food, and animal isolate data are available, and no routine sequencing has been implemented in France thus far. Therefore, this analysis was limited to clinical isolates. Increasing the number of nonclinical isolates and associated metadata would provide greater insight into the genomic diversity of circulating STEC isolates in France and enable exploration of potential transmission chains and links with clinical isolates. Those links will be particularly relevant because certain geographic zones have shown greater risk for sporadic pediatric HUS, including WGS clusters with no identified epidemiologic link (*3*).

Although WGS provides a major advance for foodborne pathogen surveillance, epidemiologic data remain essential for confirming a common source for WGS-linked isolates (*36*). This study provides insight into the diversity of situations faced by epidemiologists after introduction of WGS. Indeed, a prior study described the complexity of interpreting WGS data in light of the effects of pathogen interactions with host and reservoir and the multiple transmission mechanisms involved in STEC circulation and contamination (*36*). Within HC5 clusters, the AD and SNP distributions were variable for a single serotype and between serotypes. Although some HC5 clusters linked to point-source outbreaks had low genomic diversity, others did not, particularly O157:H7, which was historically the predominant serotype in France before 2015 (*1*). The relationship between genomic and temporal distances within HC5 clusters also illustrates that variability. Although we observed an overall positive association, we noted a negative relationship between AD and temporal distance <5 days for O26:H11 and O157:H7. That relationship could be because of a limited number of point-source outbreaks linked to a diversity of food vehicles (vegetables, raw milk cheeses, industrial frozen pizzas) and caused by strains that accumulated greater genomic diversity before the outbreak (e.g., in reservoirs, in the manufacturing ingredients or environment). Different manufacturing processes for primary and final ingredients may also contribute (initial inoculum, bottlenecks, duration of processing or aging, temperature, stress) (*37*). Periodically assessing methods of WGS cluster determination, particularly HC5, used in surveillance approaches to ensure their continued validity will be needed.

During 2018–2022, epidemiologists in France regularly investigated WGS-linked isolates with case-patients closely related in space or time, but with no common source suspected despite extensive case interviews. Although we know of inherent limitations to epidemiologic investigations (interview based, memory bias), such clusters are necessary for documenting experiences with WGS in STEC surveillance and outbreak investigations. Similar to findings reported previously, most of the HC5 clusters from France are small (<5 isolates) (*2*). Limited public health resources are directed toward investigation of larger clusters or those including severe clinical manifestations such as HUS. However, even when very small clusters are investigated, identifying a common source of contamination can be challenging because of limited epidemiologic or traceback data. Moving toward systematic documentation of epidemiologic information for all WGS-linked isolates would provide more complete data to explore and interpret relatedness but would require evolutions in prioritization of activities or additional human resources. Finally, the numerous HC5 clusters comprising isolates over several years show that, as time progresses, genomic proximity evolves to different degrees, reinforcing that a SNP-based analysis remains an essential confirmatory step for cluster determination. Threshold-based approaches, although appropriate for screening in some serotypes, may therefore not be universally applicable for a given pathogen (*12*,*38*). Overall, public health professionals should strike a balance between consideration of serotype-related limits and the advantages conferred through more standardized genomic approaches. STEC surveillance protocols on the basis of WGS data should integrate regular assessment to ensure continued validity of genomic approaches.

In summary, after 5 years of implementation of WGS for STEC surveillance, our results validate the current approach of using cgMLST HC5 as a screening step for cluster detection for 3 major serotypes in France. For the fourth major serotype, O80:H2, our results indicate that HC5 is less reliable. Regular assessment of WGS-based STEC surveillance protocols to document the effects of serotype and time (genomic evolution) is appropriate. Exploring possibilities for routinely collecting epidemiologic data for WGS clusters could enrich the capacity to describe the relationship between WGS-linked isolates and epidemiologic links.

Appendix 1 Additional information from 5 years of routine whole-genome sequencing for epidemiologic surveillance of Shiga toxin–producing *Escherichia coli*, France, 2018–2022.

Appendix 2 Information on isolates used for 5 years of routine whole-genome sequencing for epidemiologic surveillance of Shiga toxin–producing *Escherichia coli*, France, 2018–2022.
