# Supplementary material for: Lessons from 5 Years of Routine Whole-Genome Sequencing for Epidemiologic Surveillance of Shiga Toxin–Producing Escherichia coli, France, 2018–2022
Source: Emerg Infect Dis. 2025 May;31(Suppl 1):S117–28. doi: 10.3201/eid3113.241950 (PMC12078545; doi:10.3201/eid3113.241950)
Supplement: Appendix 1 — Additional information from 5 years of routine whole-genome sequencing for epidemiologic surveillance of Shiga toxin–producing Escherichia coli, France, 2018–2022. [file 24-1950-Techapp-s1.pdf]

Article DOI: https://doi.org/10.3201/eid3113.241950

EID cannot ensure accessibility for supplementary materials supplied by authors. Readers who have difficulty accessing supplementary content should contact the authors for assistance.

Lessons from 5 Years of Routine Whole-Genome Sequencing for Epidemiologic Surveillance of Shiga Toxin–Producing *Escherichia coli*, France, 2018–2022

Appendix 1

Supplemental Methods

STEC Surveillance and Outbreak Detection in France

WGS provides the highest level of case discrimination based on genomic proximity of isolates, but a strong temporal or geographic link can also suggest a common source of infection when WGS is unavailable or pending (*1*). Notification of pediatric STEC-HUS cases by physicians occurs at time of hospitalization and are the earliest signal for cluster detection (for example, an increase in STEC-HUS cases notified in a given geographic zone and period). Thus, cluster detection from physician notification data are the most sensitive, but least specific, because it does not include any discriminating microbiological data. Initial serogroup and virulence factor data from NRC-RD analysis of stool samples are typically available in the 7–10 days after hospitalization. However, timeliness of NRC-RD data depends on hospital laboratory analysis times and transport to the NRC-RD, as well as the facility of strain isolation at the NRC-RD. Although serogroup data can be discriminating in certain situations, for example, disproving a suspected cluster by identifying isolates of different serogroups or strengthening suspicions in cases of a rare serogroup isolated from several cases, more than half of pediatric STEC-HUS cases in France are infected with STEC O26 or O80 (*2*,*3*). For those predominant serogroups, WGS data are essential for greater isolate discrimination. The NRC-RD transfers all isolates to the NRC-ESS, which performs WGS weekly. Results are available for surveillance purposes around 10 days after receiving the isolates. Total turnaround time from notification of pediatric

STEC-HUS by physicians (or from sampling date for samples of other forms of infection sent to the NRC-RD) to WGS results is 3–4 weeks.

Genomic Analysis

In current WGS protocols, STEC genomic data are submitted routinely to EnteroBase (https://enterobase.warwick.ac.uk/) with limited metadata (isolate source: human, food, etc., sampling year, country). The EnteroBase cgMLST scheme for *E. coli* describes genomic proximity based on the 2,513 genes conserved across the core genome (2,510 for O80:H2 as 3 alleles, b2544, b2545, and b2547, are absent from all isolates of this serotype) (*4*). In addition, EnteroBase analysis pipeline includes a HierCC scheme, which uses multilevel, static, cluster assignments of bacterial genomes to describe genetic diversity (*5*). In brief, HierCC uses a minimum spanning tree to assign genomes to cluster at different HC levels based on the maximum number of observed allelic differences. For *E. coli*, 13 HC are reported in order of decreasing genomic proximity: HC0, HC2, HC5, HC10, HC20, HC50, HC100, HC200, HC400, HC1100, HC1500, HC2000, and HC2350. A corresponding numerical ID is automatically assigned. New genomes are assigned to the oldest cluster in cases of equal distance with multiple clusters at a given HC. At the French NRC, the HC5 level of the HierCC scheme has been chosen for the screening of microbiological clusters belonging to the same serotype. If necessary, particularly for HC5 that are persistent over time, an additional SNP analysis using the "create SNP project" tool in the EnteroBase pipeline is used to confirm clustering and guide epidemiologic investigations. Multiple examples of transmission chains and outbreaks have been described for bacterial isolates with HC20 or less (*5*,*6*).

For this study, an additional SNP-based analysis was performed to obtain SNP distance matrices and SNP-based phylogenetic trees, as follows. Short-reads obtained after the routine procedure of the NRC-ESS, as described previously, were filtered with FqCleanER version 23.07 (https://gitlab.pasteur.fr/GIPhy/fqCleanER) with options -q 28 -l 100-p 25 to eliminate adaptor sequences and discard low-quality reads with phred scores below 28 and a length of less than 100 bp, respectively (*7*). The filtered paired-end reads were mapped onto the chromosome of different *E. coli* strains belonging to each serotype evaluated in this study: *E. coli* strain 11368 (serotype O26:H11, accession number NC_013361), *E. coli* strain MOD1-EC6881 (serotype O80:H2, accession number GCF_002520045), *E. coli* strain 12009 (serotype O103:H2, accession number NC_013353), and *E. coli* strain Sakai (serotype O157:H7, accession number

NC_002695) with Snippy version 4.6.0/BWA-MEM version 0.7.17 (https://github.com/tseemann/snippy). SNPs were called with Snippy version 4.6.0/Freebayes version 1.3.2 (https://github.com/tseemann/snippy) under these constraints: mapping quality of 30, a minimum base quality of 4, and a 75% read concordance at a locus for a variant to be reported. Genomes were annotated by using RAST to identify CRISPR regions, repeats, prophages, tRNAs, and rRNAs that were further masked before building a multisequence alignment with Snippy version 4.6.0 (*8*). Putative recombinogenic regions were detected and masked with Gubbins version 3.2.0 (default settings, except --iterations 20) to generate a final core genome alignment for each serotype, with the following number of single nucleotide variant sites: 8,191 for O26:H21; 3,949 for O80:H2; 4,904 for O103:H2; and 8,191 for O157:H7 (*9*). Finally, a maximum likelihood (ML) phylogenetic tree was built from an alignment of chromosomal SNPs, with RAxML version 8.2.12, under the GTR model, with 1,000 bootstrap values, and pairwise SNP distances were calculated with a custom in-house perl script (10). The final tree was midpoint rooted and visualized with iTOL version 6 (*11*).

Supplemental Results

Genomic Distance and Epidemiologic Characteristics of HC5 Clusters

Although several persistent HC5 clusters are observed for O157:H7, the cluster sizes were small (2–6 isolates). The range of median genomic distances remained relatively small for AD (2–8 AD) and slightly larger for SNP (7–16 SNP). Of interest, for O157:H7, 2 HC5 clusters with a wider range of genomic distances comprised outbreak-linked isolates sampled over relatively short time intervals (HC5_111396 and HC5_116498) (Appendix 2 Table 2).

A greater diversity of cluster size and duration and associated genomic distances is observed for O26:H11 and O80:H2. For O26:H11, several persistent HC5 clusters comprised point source outbreaks (HC5_65006, HC5_75047), but also isolates identified outside of those outbreak periods for which no epidemiologic link was identified. The genomic distances of the entirety of cluster isolates are larger, whereas those of isolates related to point-source outbreaks within the cluster are smaller. This is reflected in SNP trees that identify distinct clustering of outbreak-linked cases within the larger HC5 cluster (Appendix 1 Figure 5). Overall, the larger range of genomic distances are not particularly associated with the longer cluster duration for

O26:H11. However, for O80:H2, a larger range of genomic distances is observed with longer cluster duration (3–5 years vs. 1–2 years) and for HC5_35179 (AD range 0–27; SNP range 0–52). Researchers have not identified an outbreak source for O80:H2 WGS clusters investigated in France.

Several large persistent clusters are observed for O26:H11. The largest persistent cluster is HC5_65006, which comprises 44 isolates sampled from 2018 to 2021. Several outbreaks linked to the same type of soft raw cow's milk cheese, but different manufacturers, were documented in 2018 and 2020. In 2020, epidemiologic investigations also confirmed an outbreak linked to recreational lake water in the same region where the raw milk cheese incriminated in previous outbreaks is manufactured. A direct source of contamination of the lake (near farms, manure-based fertilization) was not identified. In addition to the outbreak-linked cases, isolates were also sampled sporadically over the years from patients who did not report any common exposures nor any link to previously identified sources of contamination. AD and SNP distances observed for HC5_65006 are larger between all 44 isolates (median AD = 8, median SNP = 10) compared with the smaller AD and SNP distances observed between the different outbreak-linked cases: median AD varied from 1 to 3 and median SNP varied from 2 to 6.

Cluster O26:H11 HC5_38792 comprises isolates sampled primarily from patients residing in a single administrative department (6/8 isolates) in the southeast of France over a span of 3 years. Each year, 2–3 isolates were sampled in patients over a 2- to 4-month time interval. Overall, collected epidemiologic data identified no common food exposures. In a single investigation, we strongly suspected a common environmental exposure for 2 case-patients reporting no other common exposures aside of residing in a small rural village near a farm. The geographic nearness of isolates sampled over several years suggests potential circulation of this strain in a relatively restricted geographic zone. The observed genomic distances between isolates remain relatively small (median AD = 3, median SNP = 6).

Over the study period, the only epidemiologically linked isolates identified for O80:H2 were from single family transmission or from a single patient (Appendix 2 Table 2). For the largest persistent O80:H2 HC5 cluster (HC5_35179, 35 isolates over 4 years), epidemiologic investigations were conducted in 2018 (2 case-patients) and 2019 (8 case-patients), but no common source of contamination was suspected. The AD and SNP distributions are quite large

compared with other HC5 clusters and serotypes: 0–27 AD (median AD = 10) and 0–52 SNP (median SNP = 21). As seen on the SNP tree (Figure 5), the concordance between this HC5 and clustering on the SNP tree is poor overall. Investigations of HC5_140495, an HC5 cluster with isolates sampled in a single year and showing smaller genomic distances (median AD = 4, median SNP = 7), did not identify any suspected common exposures.

References

1. Besser JM, Carleton HA, Trees E, Stroika SG, Hise K, Wise M, et al. Interpretation of whole-genome sequencing for enteric disease surveillance and outbreak investigation. Foodborne Pathog Dis. 2019;16:504–12. PubMed https://doi.org/10.1089/fpd.2019.2650

2. Bruyand M, Mariani-Kurkdjian P, Le Hello S, King LA, Van Cauteren D, Lefevre S, et al.; Réseau français hospitalier de surveillance du SHU pédiatrique. Paediatric haemolytic uraemic syndrome related to Shiga toxin-producing *Escherichia coli*, an overview of 10 years of surveillance in France, 2007 to 2016. Euro Surveill. 2019;24:1800068. PubMed https://doi.org/10.2807/1560-7917.ES.2019.24.8.1800068

3. Jones G, Mariani-Kurkdjian P, Cointe A, Bonacorsi S, Lefèvre S, Weill FX, et al. Sporadic Shiga toxin–producing *Escherichia coli*–associated pediatric hemolytic uremic syndrome, France, 2012–2021. Emerg Infect Dis. 2023;29:2054–64. PubMed https://doi.org/10.3201/eid2910.230382

4. Achtman M, Zhou Z, Charlesworth J, Baxter L. EnteroBase: hierarchical clustering of 100 000s of bacterial genomes into species/subspecies and populations. Philos Trans R Soc Lond B Biol Sci. 2022;377:20210240. PubMed https://doi.org/10.1098/rstb.2021.0240

5. Zhou Z, Charlesworth J, Achtman M. HierCC: a multi-level clustering scheme for population assignments based on core genome MLST. Bioinformatics. 2021;37:3645–6. PubMed https://doi.org/10.1093/bioinformatics/btab234

6. Zhou Z, Alikhan NF, Mohamed K, Fan Y, Achtman M; Agama Study Group. The EnteroBase user's guide, with case studies on *Salmonella* transmissions, *Yersinia pestis* phylogeny, and *Escherichia* core genomic diversity. Genome Res. 2020;30:138–52. PubMed https://doi.org/10.1101/gr.251678.119

7. Jones G, Lefèvre S, Donguy MP, Nisavanh A, Terpant G, Fougère E, et al. Outbreak of Shiga toxin-producing *Escherichia coli* (STEC) O26 paediatric haemolytic uraemic syndrome (HUS) cases

associated with the consumption of soft raw cow's milk cheeses, France, March to May 2019. Euro Surveill. 2019;24:1900305. PubMed https://doi.org/10.2807/1560-7917.ES.2019.24.22.1900305

8. Aziz RK, Bartels D, Best AA, DeJongh M, Disz T, Edwards RA, et al. The RAST server: rapid annotations using subsystems technology. BMC Genomics. 2008;9:75. PubMed https://doi.org/10.1186/1471-2164-9-75

9. Croucher NJ, Page AJ, Connor TR, Delaney AJ, Keane JA, Bentley SD, et al. Rapid phylogenetic analysis of large samples of recombinant bacterial whole genome sequences using Gubbins. Nucleic Acids Res. 2015;43:e15. PubMed https://doi.org/10.1093/nar/gku1196

10. Stamatakis A. RAxML version 8: a tool for phylogenetic analysis and post-analysis of large phylogenies. Bioinformatics. 2014;30:1312–3. PubMed https://doi.org/10.1093/bioinformatics/btu033

11. Letunic I, Bork P. Interactive Tree of Life (iTOL) v5: an online tool for phylogenetic tree display and annotation. Nucleic Acids Res. 2021;49(W1):W293–6. PubMed https://doi.org/10.1093/nar/gkab301

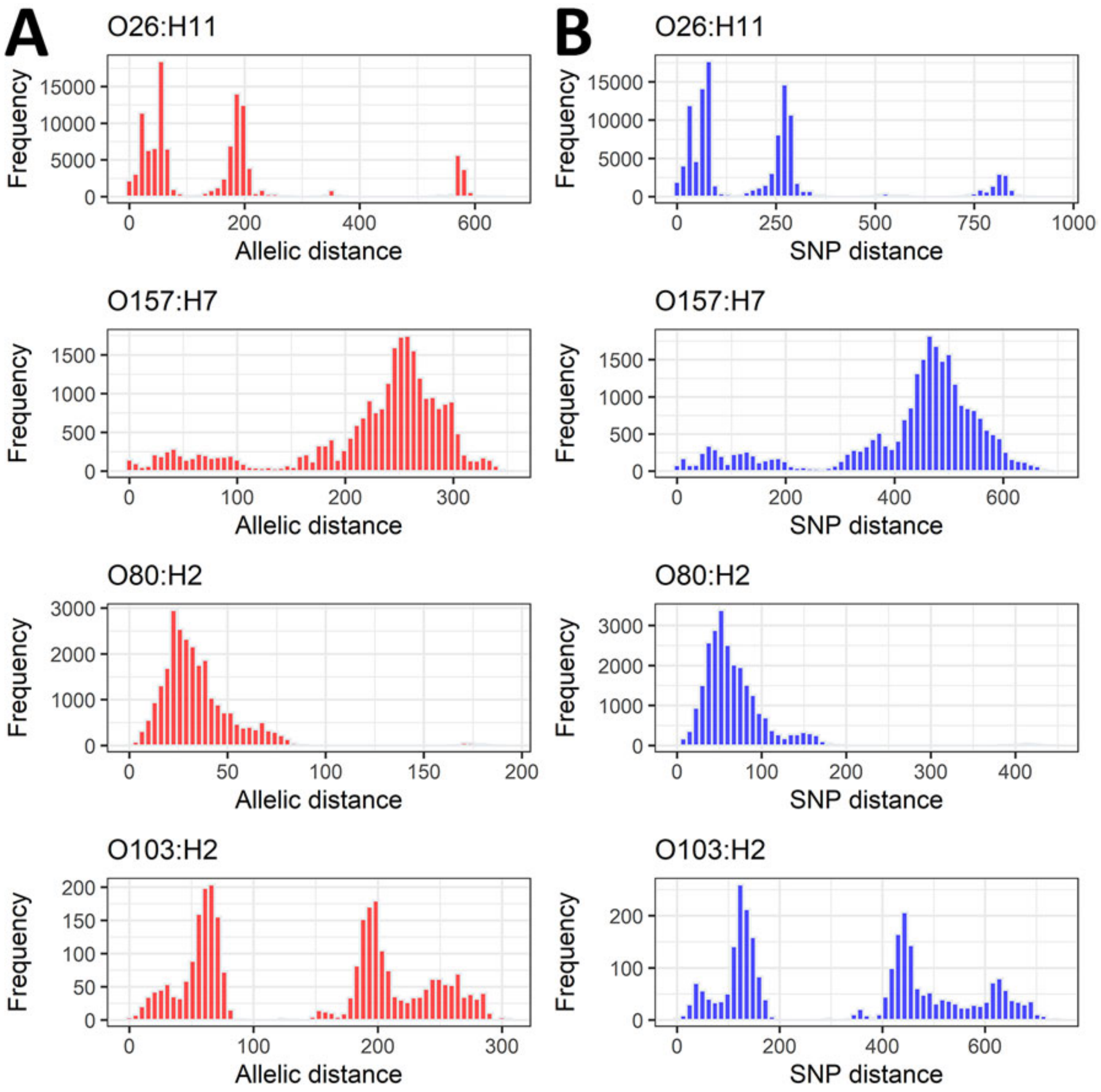

Appendix 1 Figure 1. A, B) Full distributions of pairwise allelic distances (A) and single-nucleotide polymorphism distances (B) of Shiga toxin–producing *Escherichia coli* isolated in France from 2018 to 2022. SNP, single-nucleotide polymorphism.

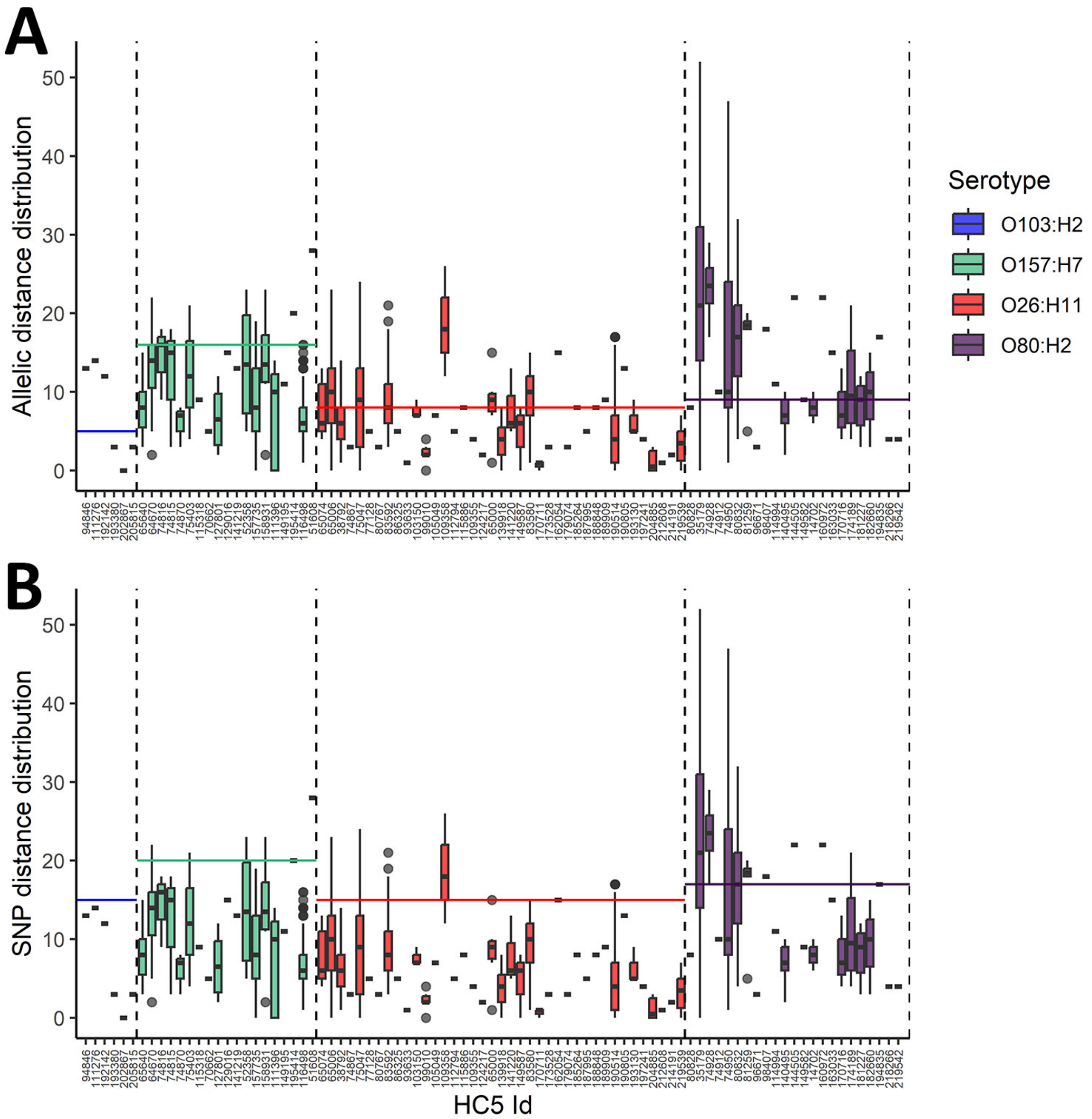

Appendix 1 Figure 2. Distance and distributions from hierarchical clustering at a threshold of 5 allelic differences from 5 years of routine whole-genome sequencing for epidemiologic surveillance of Shiga toxin–producing *Escherichia coli*, France, 2018–2022. A) Allelic distance; B) single-nucleotide polymorphism distributions. Shiga toxin–producing *Escherichia coli* serotypes are shown for each panel. Horizontal lines correspond to statistical thresholds and vertical dashed lines delineate serotypes. HC5, hierarchical clustering at a threshold of 5 allelic differences; SNP, single-nucleotide polymorphism.

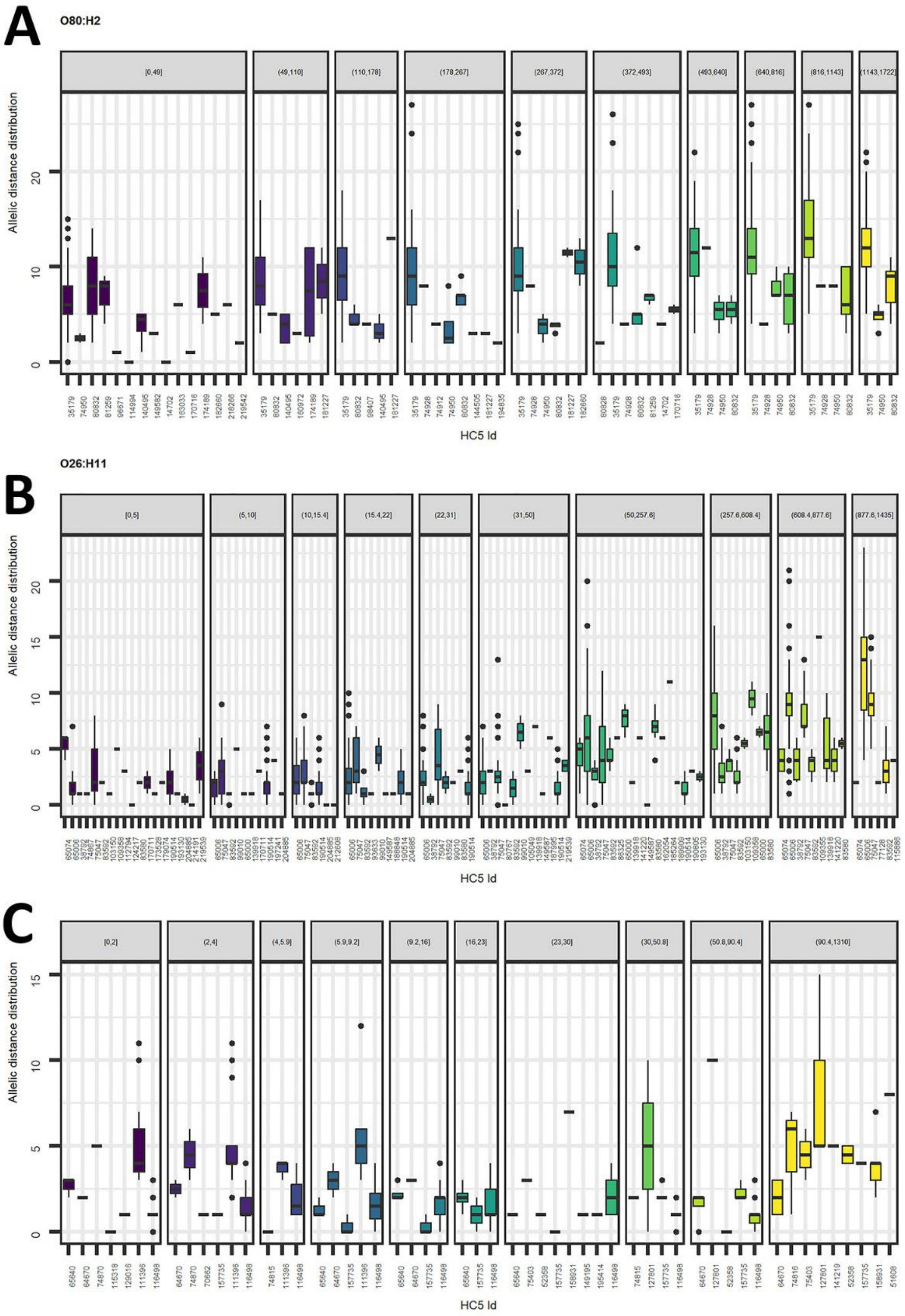

Appendix 1 Figure 3. Distributions of allelic distance within clusters of Shiga toxin–producing *Escherichia coli* identified by hierarchical clustering at a threshold of 5 allelic differences, according to time classified by decile, France, 2018–2022. Serotype O103:H2 is not shown because of limited data. HC5, hierarchical clustering at a threshold of 5 allelic differences.

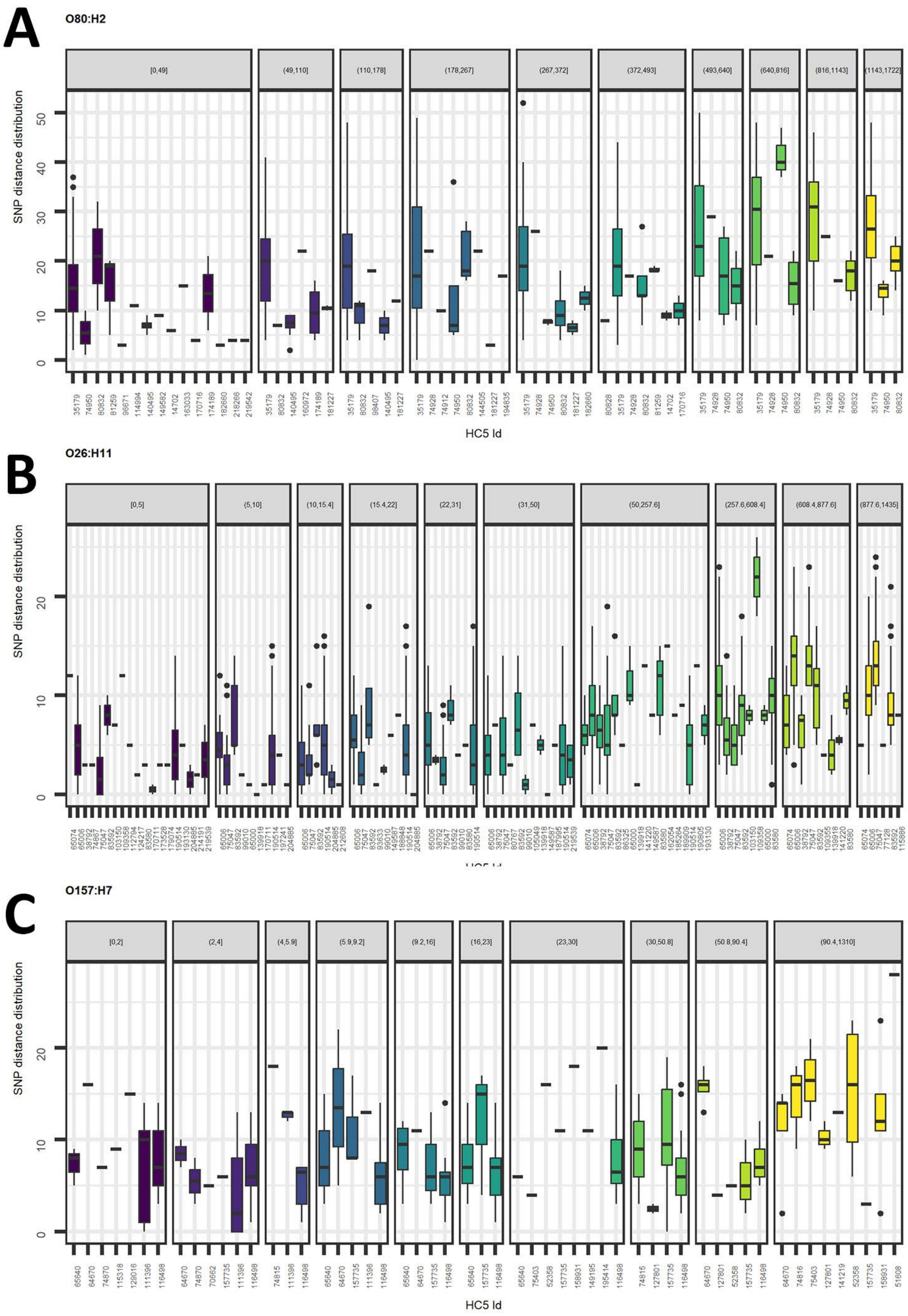

Appendix 1 Figure 4. Distributions of single-nucleotide polymorphism distances within clusters of Shiga toxin–producing *Escherichia coli* identified by hierarchical clustering at a threshold of 5 allelic differences, according to time classified by decile, France, 2018–2022. Serotype O103:H2 is not shown because of limited data. SNP, single-nucleotide polymorphism.

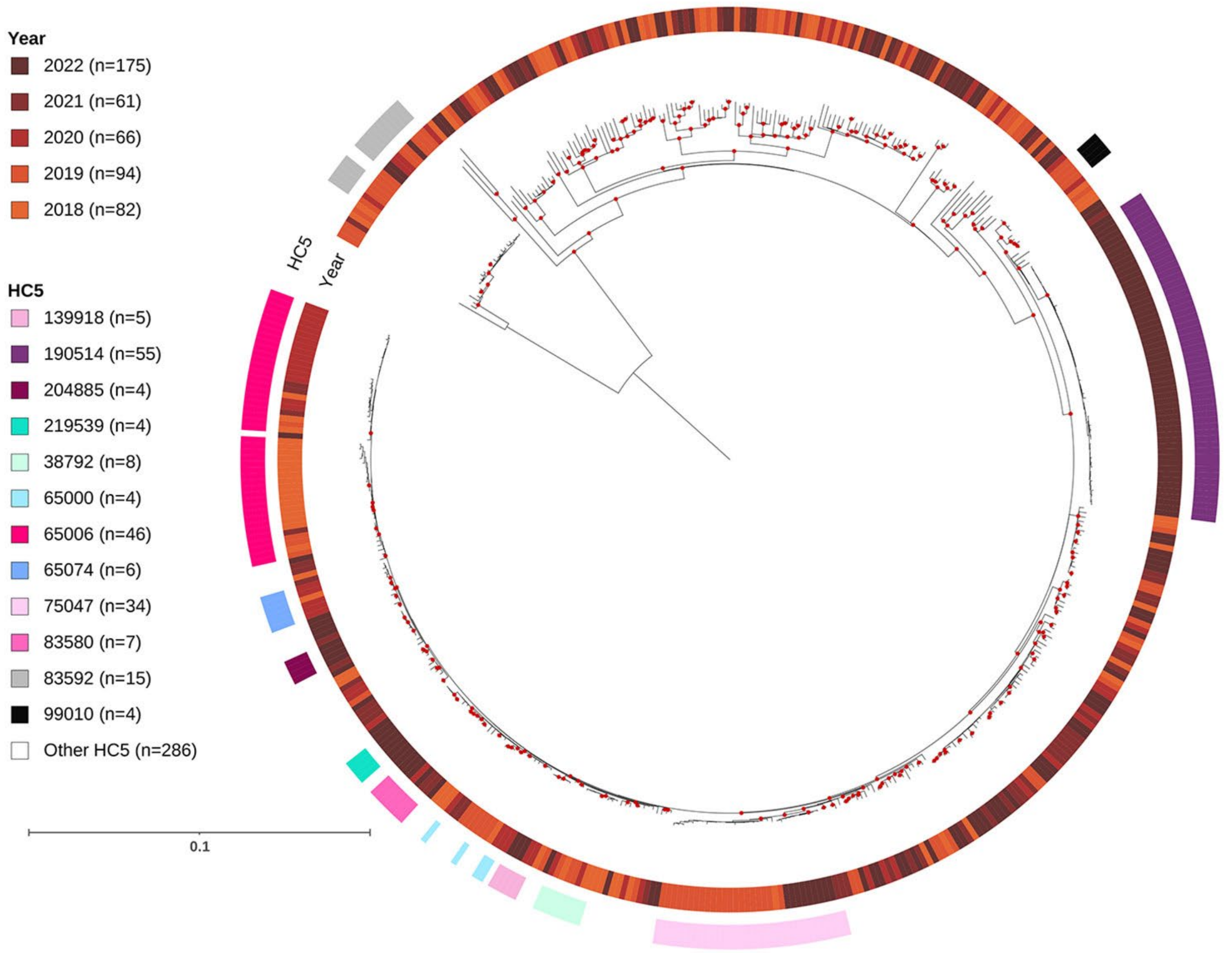

Appendix 1 Figure 5. Single-nucleotide polymorphism–based maximum likelihood phylogenetic tree of 478 Shiga toxin–producing *Escherichia coli* O26:H11 isolates from France, 2018–2022. Tree was built based on the sequence alignment of 8,191 single nucleotide variant sites of the recombination-free core genome of *E. coli* strain 11368 (accession no. NC_013361). Tree was midpoint-rooted and visualized with iTOL. Bootstrap support values ≥90% are indicated with red dots on the branches. Branch lengths and corresponding scale bar indicate the numbers of SNP per base of the final alignment. HC5, hierarchical clustering at a threshold of 5 allelic differences.

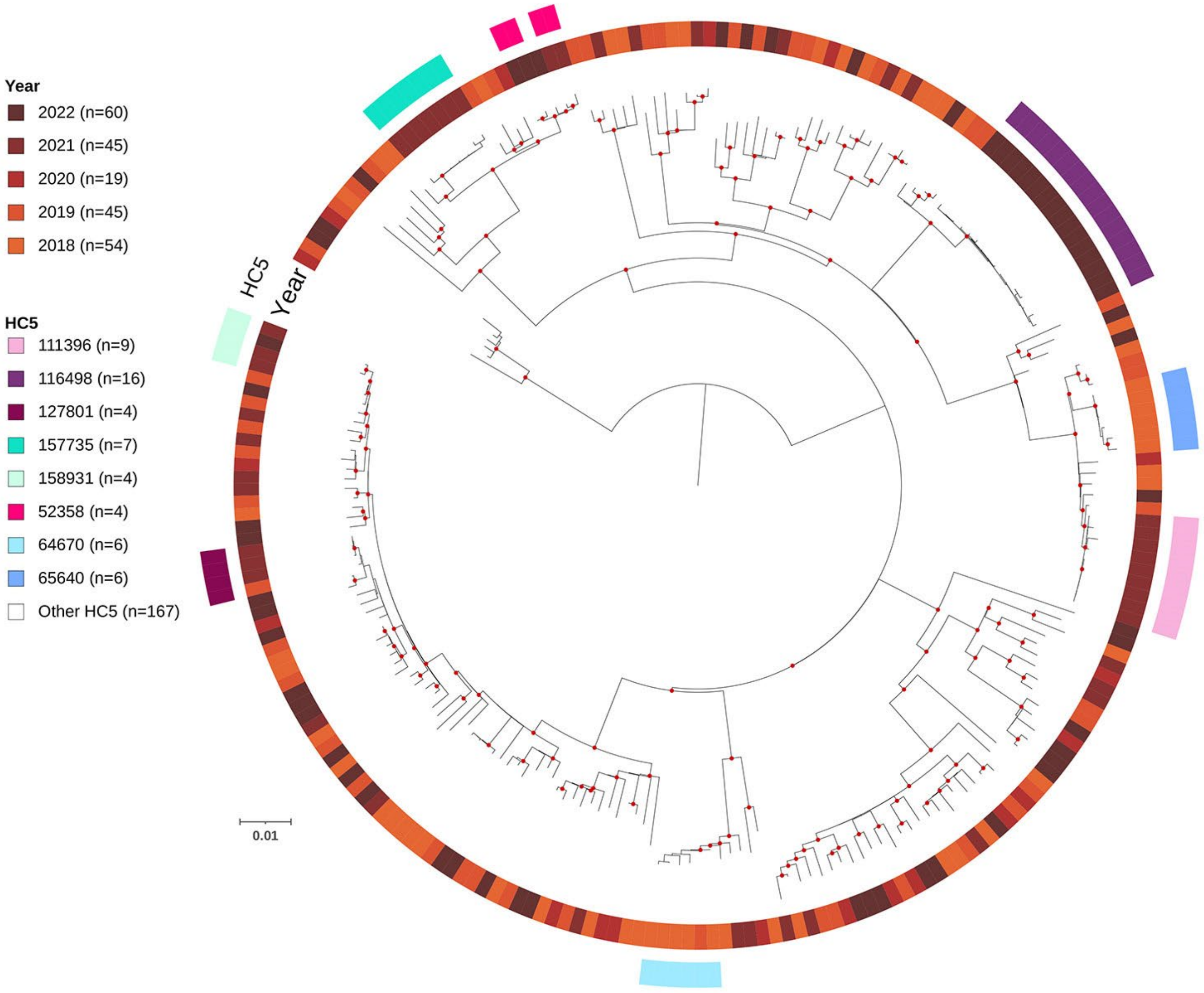

Appendix 1 Figure 6. Single-nucleotide polymorphism–based maximum likelihood phylogenetic tree of 223 Shiga toxin–producing *Escherichia coli* O157:H7 isolates from France, 2018–2022. Tree was built based on the sequence alignment of 8,191 single nucleotide variant sites of the recombination-free core genome of *E. coli* strain Sakai (accession no. NC_002695). Tree was midpoint-rooted and visualized with iTOL. Bootstrap support values ≥90% are indicated with red dots on the branches. Branch lengths and corresponding scale bar indicate the numbers of SNP per base of the final alignment. HC5, hierarchical clustering at a threshold of 5 allelic differences.

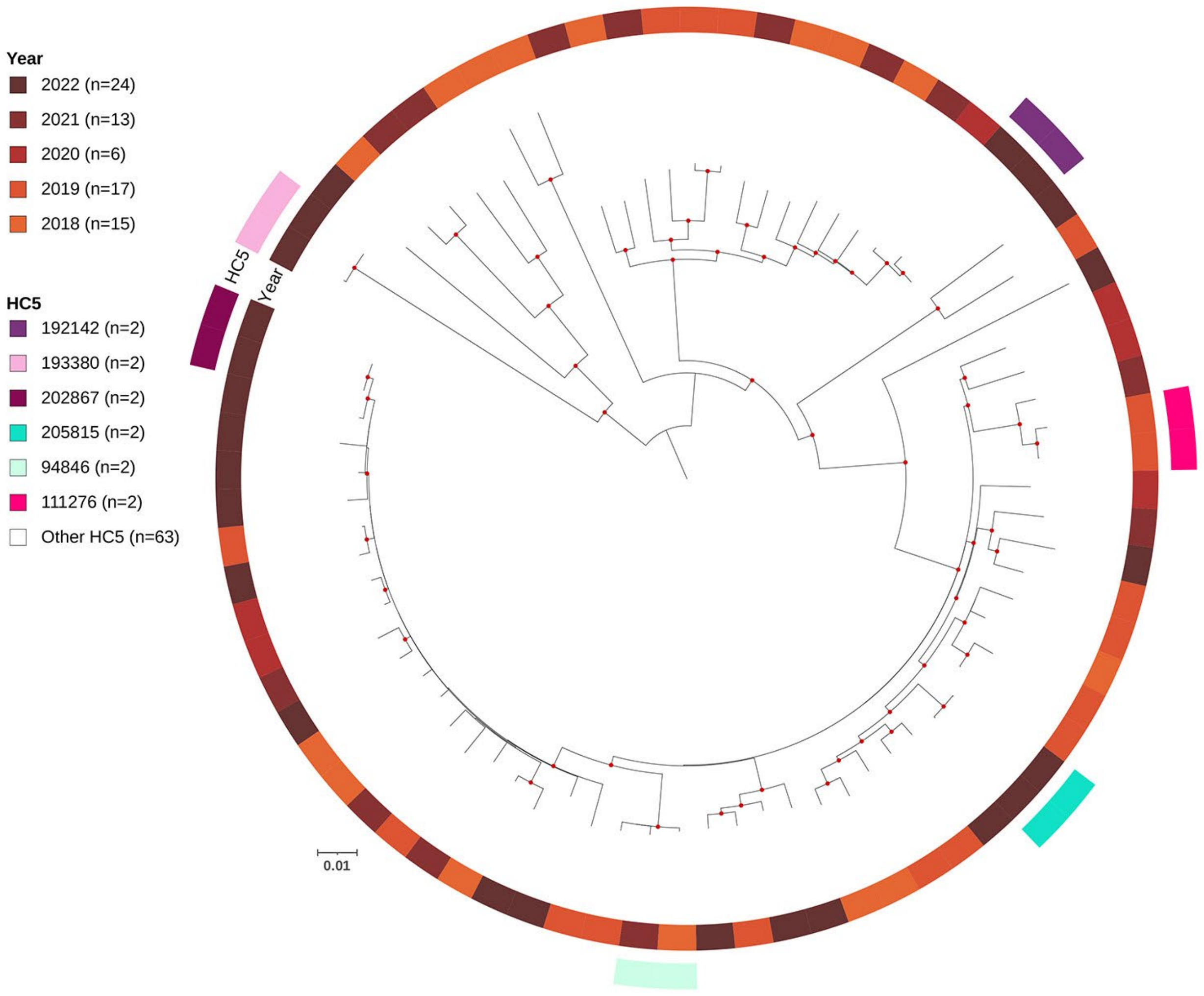

Appendix 1 Figure 7. Single-nucleotide polymorphism–based maximum likelihood phylogenetic tree of 75 Shiga toxin–producing *Escherichia coli* O103:H2 isolates from France, 2018–2022. Tree was built based on the sequence alignment of 4,904 single nucleotide variant sites of the recombination-free core genome of *E. coli* strain 12009 (accession no. NC_013353). Tree was midpoint-rooted and visualized with iTOL. Bootstrap support values ≥90% are indicated with red dots on the branches. Branch lengths and corresponding scale bar indicate the numbers of SNP per base of the final alignment. HC5, hierarchical clustering at a threshold of 5 allelic differences.
